# A Bifunctional Hybrid Electrocatalyst for Oxygen Reduction and Oxygen Evolution Reactions: Nano-Co_3_O_4_-Deposited La_0.5_Sr_0.5_MnO_3_ via Infiltration

**DOI:** 10.3390/molecules26020277

**Published:** 2021-01-08

**Authors:** Seona Kim, Guntae Kim, Arumugam Manthiram

**Affiliations:** 1 Materials Science and Engineering Program & Texas Materials Institute, The University of Texas at Austin, Austin, TX 78712, USA; seona0623@gmail.com; 2 Department of Energy Engineering, Ulsan National Institute of Science and Technology (UNIST), Ulsan 44919, Korea

**Keywords:** bifunctional catalyst, hybrid catalyst, oxygen reduction reaction, oxygen evolution reaction, four-electron pathway

## Abstract

For rechargeable metal–air batteries, which are a promising energy storage device for renewable and sustainable energy technologies, the development of cost-effective electrocatalysts with effective bifunctional activity for both oxygen reduction reaction (ORR) and oxygen evolution reaction (OER) has been a challenging task. To realize highly effective ORR and OER electrocatalysts, we present a hybrid catalyst, Co_3_O_4_-infiltrated La_0.5_Sr_0.5_MnO_3-*δ*_ (LSM@Co_3_O_4_), synthesized using an electrospray and infiltration technique. This study expands the scope of the infiltration technique by depositing ~18 nm nanoparticles on unprecedented ~70 nm nano-scaffolds. The hybrid LSM@Co_3_O_4_ catalyst exhibits high catalytic activities for both ORR and OER (~7 times, ~1.5 times, and ~1.6 times higher than LSM, Co_3_O_4_, and IrO_2_, respectively) in terms of onset potential and limiting current density. Moreover, with the LSM@Co_3_O_4_, the number of electrons transferred reaches four, indicating that the catalyst is effective in the reduction reaction of O_2_ via a direct four-electron pathway. The study demonstrates that hybrid catalysts are a promising approach for oxygen electrocatalysts for renewable and sustainable energy devices.

## 1. Introduction

Renewable and sustainable energy storage and conversion systems such as fuel cells, electrolysis cells, and secondary batteries have attracted a great deal of attention in recent years to address the environmental challenges we face today [1,2,3,4]. Specifically, metal–air batteries are considered to be promising systems due to their extremely high theoretical energy density [4,5,6,7,8,9]. Metal–air batteries consist of a pure metal (e.g., Li, Na, Zn, Al) anode and an external cathode of ambient air with an aqueous or aprotic electrolyte. To be suitable for rechargeable batteries, the oxygen electrocatalysts should exhibit effective catalytic activity, not only for the oxygen reduction reaction (ORR), but also for the oxygen evolution reaction (OER)—that is, they should exhibit bifunctionality [10,11,12,13,14,15]. However, due to the sluggish reaction kinetics of the ORR and OER (which respectively correspond to the discharge and charge processes), the development of highly efficient and cost-effective oxygen electrocatalysts is considered to be an essential challenge. Although Pt, IrO_2_, RuO_2_, and their alloys and composites show superior activities for ORR or OER, they suffer from the drawbacks of poor stability, scarcity, and high cost. 

The focus in the literature has been on transition-metal oxides because oxygen electrocatalysts possess bifunctionality due to their operational stability in alkaline solutions and promising catalytic activities for ORR and OER. Among them, Co-containing oxides such as Co_3_O_4_, Ln*_x_*Sr_1-*x*_CoO_3-*δ*_, and Ba*_x_*Sr_1-*x*_Co*_y_*Fe_1-*y*_O_3-*δ*_ have especially been reported as efficient electrocatalysts for OER [13,16,17,18,19,20,21]. Mn-containing oxides (e.g., Mn_2_O_3_, NiMnO_2_, CoMnO_2_, and La_0.5_Sr_0.5_MnO_3-*δ*_) have demonstrated excellent catalytic activity for ORR [22,23,24,25,26]. Although the above transition-metal oxides exhibit some bifunctionality, their outstanding catalytic activity is generally specific to either ORR or OER, but not both. 

Hybrid catalysts composed of more than two materials have been suggested as an approach to secure the desired bifunctionality. For example, Cu-nanoparticle-loaded Co_3_O_4_ microspheres, Co_3_O_4_–graphene composite, LaNiO_3_-Pt/C core–corona structure catalyst, and Co_3_O_4_ nanocrystals grown on graphene have been demonstrated [8,17,27,28,29,30,31]. Based on earlier studies, hybrid catalysts (A@B) show enhanced ORR/OER activity compared to the individual catalysts (A or B) and/or their physical mixtures (A + B) due to the synergistic effects between the constituent materials [17,27,32]. Therefore, in this study, a hybrid catalyst consisting of La_0.5_Sr_0.5_MnO_3-*δ*_ (LSM) and Co_3_O_4_ was designed to achieve high catalytic activities for both ORR and OER. 

Nanosizing fabrications were employed to maximize the performances of each constituent material by endowing enlarged reaction sites and shortened ion-/charge-transport path [33,34,35]. Among the various fabrication techniques, electrospray and infiltration techniques have been used due to their simplicity, effectiveness, and reproducibility. In addition, the stoichiometric ratios can be precisely controlled because the fabrication techniques rely on the precursor mixing at the molecular level. 

LSM nanoparticles were prepared through electrospray, and Co_3_O_4_ was deposited on the LSM surface through infiltration to synthesize the hybrid catalyst (LSM@Co_3_O_4_). We discovered that 20 wt.% Co_3_O_4_-infiltrated LSM exhibited a comparable catalytic activity to benchmark bifunctional oxygen electrocatalysts. Furthermore, because the calculated number of transferred electrons is four, it indicates that O_2_ follows a direct reduction pathway with less thermodynamic reaction potential. 

## 2. Results and Discussion

### 2.1. Structural and Morphological Characterization

**Figure 1:**
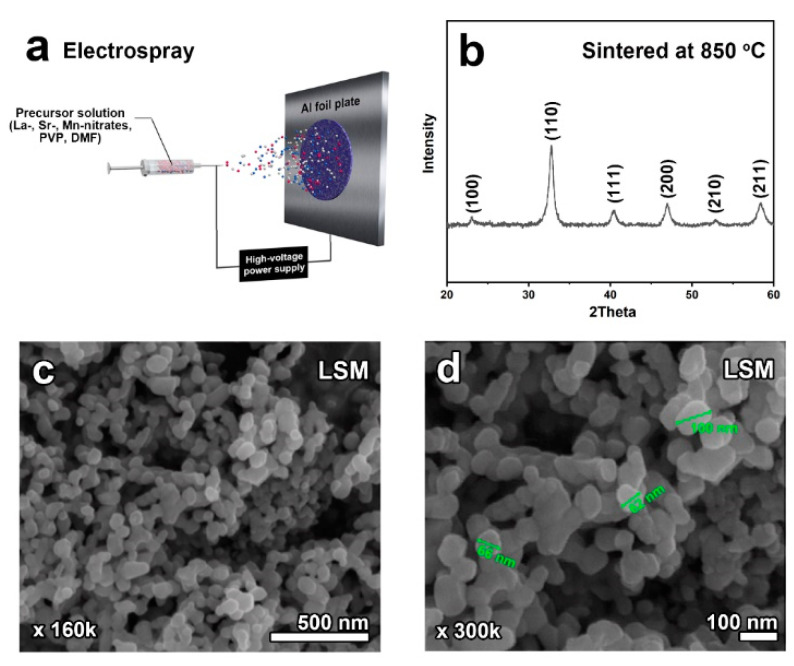
(**a**) Schematic illustration of the electrospray fabrication of La_0.5_Sr_0.5_MnO_3-*δ*_ (LSM). (**b**) X-ray diffraction patterns and (**c**,**d**) scanning electron microscopy images of LSM sintered at 850 °C for 4 h in air.

Figure 1a illustrates the electrospray fabrication of La_0.5_Sr_0.5_MnO_3-*δ*_ (LSM) with the precursor solution including La-, Sr-, and Mn nitrates, PVP, and DMF. A syringe needle tip and aluminum foil collector were connected to the anode and cathode of a high-voltage power supply, respectively. After a heat treatment at 850 °C, X-ray diffraction (XRD) was conducted in the range of 20° < 2θ < 60° to identify the crystal structure of the prepared samples. As shown in Figure 1b, LSM was successfully synthesized with a single-phase structure of simple perovskite without any significant impurities. The scanning electron microscopy (SEM) images in Figure 1c, d illustrate that spherical LSM nanoparticles 60–100 nm in size were formed and organically connected.

**Figure 2:**
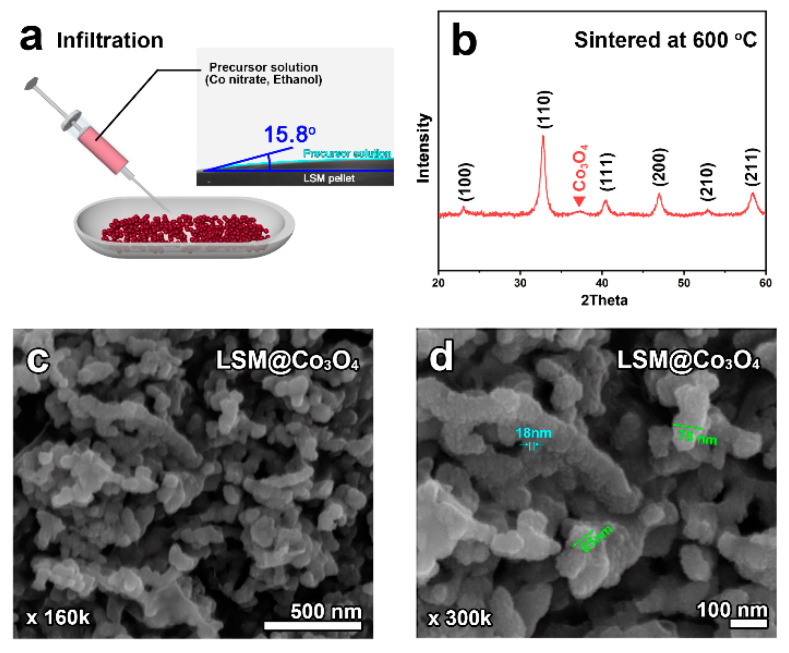
(**a**) Schematic illustration of the infiltration technique for LSM@Co_3_O_4_ with the contact angle of the precursor solution (inset). (**b**) X-ray diffraction pattern and (**c**,**d**) scanning electron microscopy images of LSM@Co_3_O_4_ sintered at 600 °C for 4 h in air.

Employing the infiltration technique for these nanoparticle scaffolds is a new approach and a unique attempt. Typically, micro-scale pore/particles have been used as the scaffold for infiltration technique and the nanoparticles deposited on the scaffold surface have a size of 60 nm or more. However, the nano-sized LSM scaffold has low interactive force with the precursor solution due to the reduced contact area. To overcome this problem and increase the wettability, ethanol was used as the solvent in this study due to its low boiling point, low surface tension (22.3 mN m^−1^ at 20 °C), and good solubility of cobalt nitrate. The contact angle of the precursor solution was measured to check the wettability by dropping 3 μL of the precursor solution on the polished LSM pellet. The cobalt precursor solution had a low contact angle of 15.8° (inset in Figure 2a), which indicates good wettability, and the use of ethanol facilitated the deposition of precursors on the surface of LSM nanoparticles. Table 1 summarizes the abbreviations for the samples.

To obtain an effective Co_3_O_4_ electrocatalyst, the infiltrated powder was sintered at 600 °C and the sintered powder is denoted as LSM@Co_3_O_4_. The XRD pattern of LSM@Co_3_O_4_ (Figure 2b) shows the phase of LSM and Co_3_O_4_ without any detectable impurity phases, indicating the successful synthesis of Co_3_O_4_. The broad peak due to Co_3_O_4_ can be attributed to the nano size of the deposited particles. The high-magnification SEM images of LSM@Co_3_O_4_ in Figure 2c,d confirm that the smaller nanoparticles (~18 nm) of Co_3_O_4_ were uniformly deposited on the entire LSM nano-scaffold (~70 nm). Furthermore, LSM@Co_3_O_4_ retained the organically connected microstructures, which is effective for the transfer of electrons.

### 2.2. Rotating Ring-Disk Electrode (RRDE) Test

**Figure 3:**
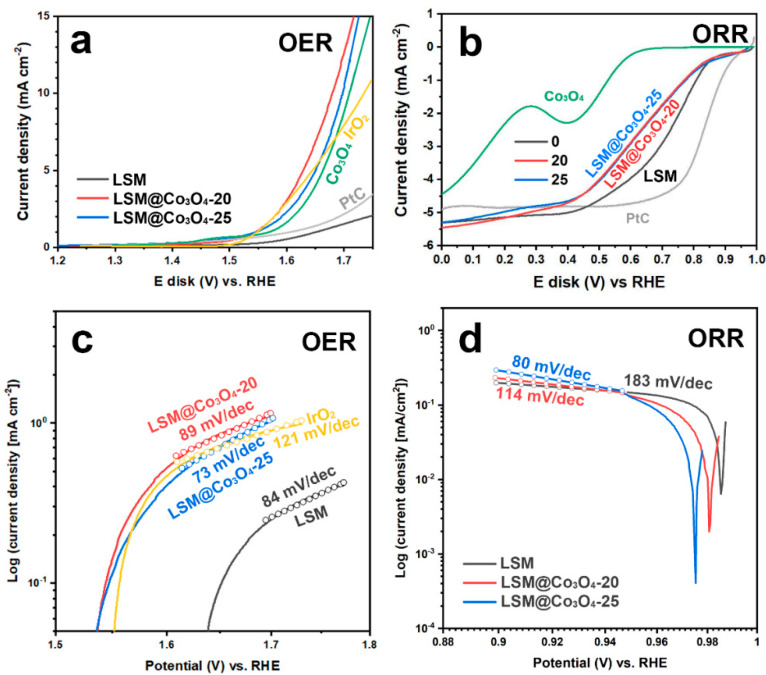
Polarization curves and Tafel slopes according to the loading amount of Co_3_O_4_ for (**a**,**c**) oxygen evolution reaction (OER) and (**b**,**d**) oxygen reduction reaction (ORR) in O_2_-saturated 0.1 M KOH at a rotation rate of 1600 rpm and a scan rate of 0.01 V s^−1^.

With different loading amounts of Co_3_O_4_, the catalytic activities of LSM@Co_3_O_4_ were measured for OER and ORR in 0.1 M KOH. The catalytic activities were compared with the benchmark catalysts IrO_2_ and Pt/C for OER and ORR to evaluate LSM@Co_3_O_4_ as a bifunctional electrocatalyst. For OER, the catalytic activities of LSM, LSM@ Co_3_O_4_-20, and LSM@Co_3_O_4_-25 were compared with Co_3_O_4_ and the benchmark catalyst IrO_2_ (Figure 3a). LSM has a nano-sized microstructure, but its OER current density showed a low value of 1.85 mA cm^−2^ at 1.70 V vs. reversible hydrogen electrode (RHE). After conducting Co_3_O_4_ infiltration, the current density was significantly enhanced to 13.01 mA cm^-2^ for LSM@Co_3_O_4_-20 and 10.63 mA cm^-2^ for LSM@Co_3_O_4_-25, indicating that the surface material was the dominant factor determining the catalytic activity for the OER. Especially, the current density of LSM@Co_3_O_4_-20 was ~7 times, ~1.5 times, and ~1.6 times higher, respectively, than those of LSM, Co_3_O_4_, and IrO_2_. The onset potential was also enhanced, as could be deduced from the voltage at 1 mA cm^-2^ of LSM@Co_3_O_4_-20 (1.55 V) and LSM@Co_3_O_4_-25 (1.55 V), compared to those of LSM (1.65 V) and Co_3_O_4_ (1.57 V). Moreover, the values are comparable to those of benchmark electrocatalysts such as Pt/C (1.61 V) and IrO_2_ (1.55 V). To compare OER kinetics, Tafel plots of LSM, LSM@Co_3_O_4_-20, and LSM@Co_3_O_4_-25 were derived from their OER polarization curves, and are presented in Figure 3c. The Tafel slope of LSM@Co_3_O_4_-25 was 73 mV decade^-1^, which is lower than that of LSM (84 mV decade^-1^), LSM@Co_3_O_4_-20 (89 mV decade^-1^), and IrO_2_ (121 mV decade^-1^). These improved catalytic activities of LSM@Co_3_O_4_-20 imply the existence of synergistic effects between LSM and Co_3_O_4_ and the potential for the use of LSM@Co_3_O_4_-20 as a bifunctional electrocatalyst.

Figure 3b,d presents the polarization curves of LSM@Co_3_O_4_ for the ORR with the benchmark samples Co_3_O_4_ and Pt/C. LSM on its own exhibited a good limiting current density (−5.30 mA cm^-2^) compared to Pt/C (−4.90 mA cm^-2^). However, with the Co_3_O_4_ infiltration, the limiting current density was enhanced to −5.45 and −5.30 mA cm^-2^, respectively, for LSM@Co_3_O_4_-20 and LSM@Co_3_O_4_-25. The ORR onset potentials at a current density of −0.3 mA cm^-2^ were observed to be 0.87, 0.87, and 0.89 V vs. RHE, respectively, for LSM, LSM@Co_3_O_4_-20, and LSM@Co_3_O_4_-25. On its own, Co_3_O_4_ showed a lower ORR activity, with the values of −4.45 mA cm^-2^ for limiting current density and 0.59 V vs. RHE for onset potential. Considering that a hybrid catalyst (A@B) has the properties of both constituent materials (A and B), the reduced activity in the range of 0.10–0.89 V may originate from the low catalytic activity of Co_3_O_4_, covering the surface of LSM. In Figure 3d, the Tafel slopes are plotted from the ORR polarization curves near the onset potential. Tafel slopes were obtained from the Koutecky–Levich (K-L) equation: LSM (80 mV decade^-1^), LSM@Co_3_O_4_-20 (114 mV decade^-1^), and LSM@Co_3_O_4_-25 (184 mV decade^-1^). The lower Tafel slope for LSM@Co_3_O_4_ compared to that for LSM indicates that the infiltration of Co_3_O_4_ facilitated oxygen diffusion on the catalyst surface and improved the ORR performance.

### 2.3. Four-Electron Pathway

**Figure 4:**
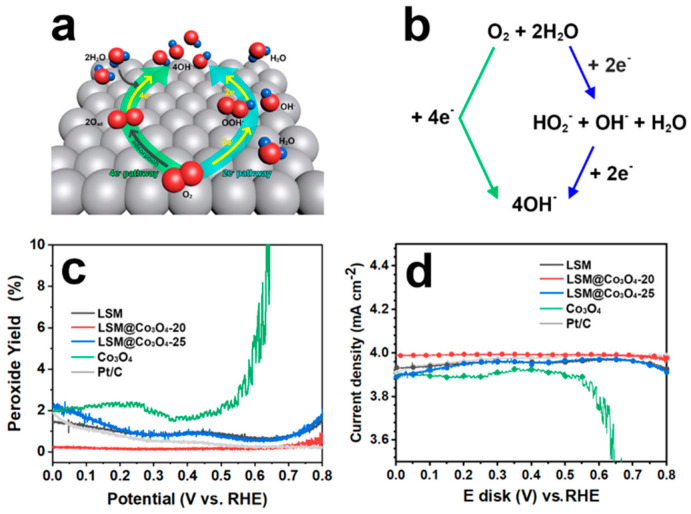
(**a**) Electron transfer number (*n*) depending on the loading amount of Co_3_O_4_ for the oxygen reduction reaction (ORR). (**b**) Schematic representation of the ORR mechanism by direct 4-electron and indirect 2+2 electron pathways. (**c**) Assessment of peroxide yield calculated by the (**d**) ring current in O_2_-saturated 0.1 M KOH at a rotation rate of 1,600 rpm and a scan rate of 0.01 V s^−1^.

As shown in Figure 4a,b, the electrochemical reduction of O_2_ can occur via two pathways, either a direct 4-electron pathway or a 2+2-electron pathway with peroxide intermediates. For oxygen electrocatalysis, the 4-electron pathway is most desirable because the direct pathway requires less thermodynamic reaction potential (Table 2). The 2+2-electron pathway is preferred in the industry for H_2_O_2_ production rather than for ORR catalysis.

To verify the ORR catalytic pathways, ring disk current was employed to detect the current of HO_2_^-^ concomitantly produced via a two-electron pathway. The peroxide yield and electron transfer number (*n*) were calculated from the following equations with the measured current of the ring electrode and disk electrode for LSM, LSM@Co_3_O_4_-20, and LSM@Co_3_O_4_-25. The values of commercial Pt/C and Co_3_O_4_ were also compared as a benchmark.

% HO_2_^-^ = 200 × (I_r_/N)/(I_d_ + I_r_/N) (1)

*n* = 4 × I_d_/(I_d_ + I_r_/N) (2)

where I_d_ represents the disk current, I_r_ indicates the ring current, and N is the current collection efficiency of the Pt ring. N was experimentally determined to be 0.41 from the reduction of K_3_Fe[CN]_6_. 

The measured H_2_O_2_ yield was below 3% for LSM, LSM@Co_3_O_4_-20, and LSM@Co_3_O_4_-25, which is comparable to that for Pt/C and much lower than that for Co_3_O_4_ (Figure 4c). The number of electrons transferred for LSM@Co_3_O_4_-20 was calculated to be 3.96–4.00 (Figure 4d), which is higher than that for LSM (3.90–3.96) and LSM@Co_3_O_4_-25 (3.90–3.97). These results confirm that LSM, LSM@Co_3_O_4_-20, and LSM@Co_3_O_4_-25 followed a 4-electron reduction pathway rather than the 2+2-electron pathway. Especially, LSM@Co_3_O_4_-20 formed a lower quantity of peroxides than Pt/C, allowing the ORR to proceed through an almost ideal 4-electron reduction pathway.

### 2.4. X-Ray Photoelectron Spectroscopy (XPS) Anaylsis

Based on the RRDE results, LSM@Co_3_O_4_ exhibited good catalytic activity for both ORR and OER, comparable to the benchmark catalysts (Pt/C, Co_3_O_4_, and IrO_2_). Since the catalytic activities of LSM@Co_3_O_4_-20 and LSM@Co_3_O_4_-25 were similar and the *n*-value of LSM@Co_3_O_4_-20 was closer to four, we selected LSM@Co_3_O_4_-20 as a representative sample of LSM@Co_3_O_4_ for X-ray photoelectron spectroscopy (XPS) characterization. 

**Figure 5:**
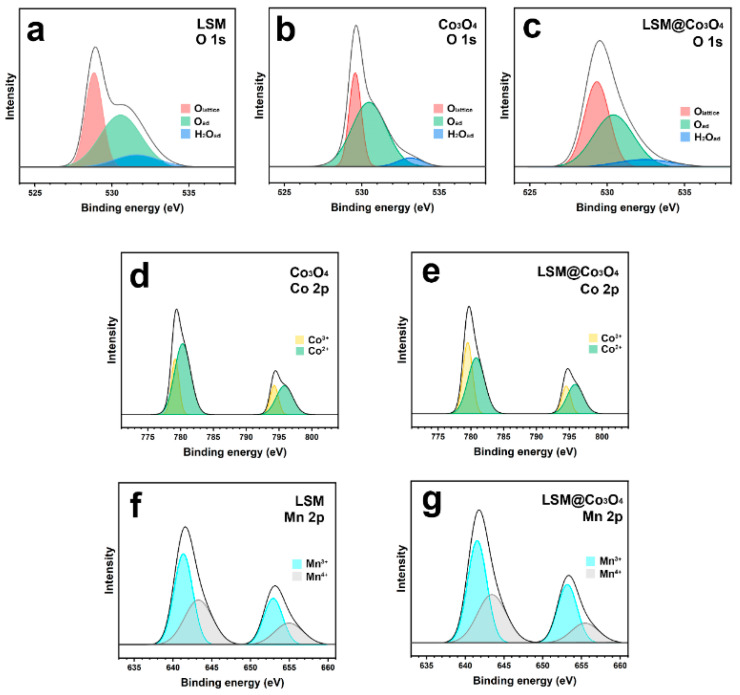
(**a**–**c**) O 1s, (**d**,**e**) Co 2p, and (**f**,**g**) Mn 2p XPS peaks of, respectively, LSM, Co_3_O_4_, and LSM@Co_3_O_4_-20.

XPS was performed to analyze the surface electronic state of the catalysts (i.e., LSM, Co_3_O_4_, and LSM@Co_3_O_4_-20) for O 1s, Co 2p, and Mn 2p. The XPS results were calibrated with the binding energy (BE) of the C 1s peak at 284.3 eV. In Figure 5a–c, the O 1s spectrum can be deconvoluted into three main peaks of lattice oxygen (O_lattice_, red), surface-adsorbed oxygen species (O_ad_, green), and adsorbed molecular water (H_2_O_ad_, blue). The relative contents of the oxygen species were calculated from the relative area of the three peaks, and are listed in Table 3. The calculated O_ad_/O_lattice_ ratios were 1.68, 2.02, and 2.39, respectively, for LSM, Co_3_O_4_, and LSM@Co_3_O_4_-20. Considering that O_ad_ can easily be converted to O_2_, the higher O_ad_/O_lattice_ ratio of LSM@ Co_3_O_4_-20 is in good agreement with the OER results (i.e., indicating an enhanced catalytic activity of LSM@ Co_3_O_4_-20).

According to previous studies, Co^3+^ cations at the surface play a significant role in the OER due to their unique electronic state, favorable as both an electron donor and an electron acceptor for O_2_ and electron capturing. The Co^3+^/Co^2+^ ratio was 0.45 for Co_3_O_4_ and 0.68 for LSM@Co_3_O_4_-20, respectively. The highly concentrated Co^3+^ of LSM@Co_3_O_4_-20 indicates the high number of donor–acceptor reduction sites, supporting the enhanced OER catalytic activity. As presented in Figure 5f,g, the Mn^3+^/Mn^4+^ ratio was reduced from 0.83 for LSM to 0.77 for LSM@Co_3_O_4_-20, indicating ligand effects between LSM and the infiltrated Co_3_O_4_ layer. The ligand effect, a type of interfacial effect that leads to synergistic effects on the catalytic activity, refers to the electron transfer derived by the different electronic configurations of two adjacent materials. It has good agreement with the XPS results for Co 2p, in which LSM@Co_3_O_4_-20 had a higher Co^3+^ concentration compared to Co_3_O_4_. Based on these results, we speculate that the electrons in the LSM layer were transferred to the infiltrated Co_3_O_4_ layer by ligand effects, resulting in an electron-rich state on the surface. Correspondingly, the bond strength between the metal oxide and oxygen species at the surface of an electrocatalyst can be altered and the catalytic activity for ORR and OER can be tuned.

## 3. Conclusions

In this study, we presented a hybrid catalyst as a bifunctional catalyst for both ORR and OER. To synthesize the hybrid catalyst, 18 nm Co_3_O_4_ nanoparticles were uniformly deposited over the entire surface of a ~70 nm LSM nano-scaffold through simple electrospray and infiltration. The hybrid catalysts exhibited comparable OER and ORR performances compared to the benchmarked commercial catalysts. In particular, the hybrid catalysts followed a four-electron pathway, meaning a very effective path to the ORR. Due to differences in the electronic configurations of the ions in Co_3_O_4_ and LSM, the concentration of Co^3+^ and electron-rich phases were increased in the hybrid catalyst, contributing to the enhancement in the catalytic activity of ORR and OER.

## 4. Materials and Methods 

La_0.5_Sr_0.5_MnO_3-*δ*_ nanoparticles were synthesized via electrospray using a precursor solution consisting of La(NO_3_)_3_·6H_2_O (99+%, Sigma Aldrich Co.), Sr(NO_3_)_2_ (99+%, Sigma Aldrich Co.), Mn(NO_3_)_2_ (98%, Sigma Aldrich Co.), poly-vinylpyrrolidone (PVP, avg. molecular weight ~1,300,000 by LS, Sigma-Aldrich Co.), and N,N-dimethylformamide (DMF, Alfa Aesar Co.). The precursor solution was prepared by mixing the metal nitrates in DMF solvent with continuous stirring. After adding the PVP to the resulting solution, it was stirred for 12 h at 60 °C. The as-prepared LSM/PVP solution was filled into a plastic syringe with a capillary tip (D = 0.5 mm), electrically connected to a high-voltage power supply from Korea switching Co. The needle tip and aluminum collector were connected to the anode and cathode of the power supply, respectively, and were placed horizontally (Figure 2a). The applied voltage was 15 kV, and the distance between the tip and aluminum foil collector was 14 cm. The collected precursor was peeled off and heated at 850 °C for 4 h at a rate of 2 °C min^-1^ in air. 

For the infiltration process, the precursor solution was prepared by dissolving Co(NO_3_)_2_·6H_2_O (98+ %, Sigma Aldrich Co.) in ethanol, which was penetrated into the LSM powder (prepared via electrospray). The resultant product was calcinated at 450 °C for 15 min in air. These penetration–calcination steps were repeated until the loading amount reached 20 and 25 wt.%. The loading amounts of Co_3_O_4_ was chosen with the consideration of interfacial effects, investigated in a previous study [12]. The samples with 20 and 25 wt.% Co_3_O_4_ are designated hereafter as LSM@Co_3_O_4_-20 and LSM@Co_3_O_4_-25, respectively. The infiltrated LSM was sintered at 600 °C for 4 h at a ramping rate of 2 °C min^-1^ in ambient air to obtain Co_3_O_4_. The loading amount was calculated using Equation (3):

Loading amount of Co_3_O_4_ (wt.%) = (W_LSM@Co3O4_ − W_LSM_)/ W_LSM_ × 100 (3)

where W_LSM_ is the weight of the LSM before infiltration and W_LSM@Co3O4_ is the weight of LSM@Co_3_O_4_ after sintering at 600 °C. 

X-ray diffraction (XRD, Bruker D8 Advance) was performed in the range of 20° < 2θ < 60° for structural analyses. Scanning electron microscopy (SEM) (Nova SEM) was conducted to observe the microstructures of LSM and Co_3_O_4_@LSM. X-ray photoelectron spectroscopy (XPS) data were acquired with ESCALAB 250XI (Thermo Fisher Scientific) with a monochromated Al-Kα (ultraviolet He1, He2) X-ray source. To evaluate the electrocatalytic activity for ORR and OER, rotating ring-disk electrode (RRDE) experiments were performed with the prepared ink composed of 20 mg of sample, 0.9 mL of solvent (ethanol: isopropyl alcohol = 1:1), and 0.1 mL of 5 wt.% Nafion solution (Aldrich, 274704). The well-dispersed catalyst ink (5 mL) was spread on the pre-polished glassy carbon (GC) disk electrode (0.1256 cm^2^). The electrochemical performances with an RRDE-3A rotating disk electrode system were recorded with a Biologic VMP3. A Pt wire, Hg/HgO electrode filled with 1 M NaOH, and 0.1 M KOH solution saturated with oxygen were respectively used as the counter electrode, reference electrode, and electrolyte. To estimate the amount of peroxide from the disk electrode during the ORR, +0.4 V was applied to the ring electrode. 

To convert the potential values from vs. Hg/HgO to vs. the reversible hydrogen electrode (RHE), the difference was measured in a cell using Pt wires for the working and counter electrodes under H_2_-saturated electrolyte 0.1 M KOH with an Hg/HgO reference electrode. The open-circuit potential was −0.89 V at 1 mV s^-1^ and the relationship between Hg/HgO and RHE was represented as:

E_Hg/HgO_ + 0.89 V = E_RHE_. (4)

## Figures and Tables

**Table 1:** Chemical compositions and abbreviations of the samples.

Abbreviations		Chemical Composition
LSM		La_0.5_Sr_0.5_MnO_3-*δ*_
LSM@Co_3_O_4_	LSM@Co_3_O_4_-20	20 wt.% Co_3_O_4_-infiltrated LSM
	LSM@Co_3_O_4_-25	25 wt.% Co_3_O_4_-infiltrated LSM

**Table 2:** Thermodynamic electrode potential of the O_2_ reduction pathway.

	Reaction Process	Thermodynamic Electrode Potential at Standard Conditions, V vs. SHE
4-electron pathway	O_2_ + 2H_2_O + 4e^-^ → 4OH^-^	0.401
2+2-electron pathway	O_2_ + H_2_O + 2e^-^ → HO_2_^-^ + OH^-^	−0.065
	HO_2_^-^ + H_2_O + 2e^-^ → 3OH^-^	0.867

**Table 3:** XPS peak deconvolution results of LSM, CO_3_O_4_, and LSM@Co_3_O_4_-20.

Sample	Species			BE (eV)	Ratio (Co^3+^/Co^2+^)	Ratio (Mn^3+^/Mn^4+^)	Ratio (O_ad_/O_lattice_)
LSM	Mn	2p_3/2_	Mn^3+^	641.49		0.83	
			Mn^4+^	643.36			
		2p_1/2_	Mn^3+^	653.26			
			Mn^4+^	656.21			
	O 1s	O_lattice_		528.77			1.68
		O_ad_		528.98			
		H_2_O_ad_		530.96			
	C 1s			284.3			
Co_3_O_4_	Co	2p_3/2_	Co^3+^	779.32	0.45		
			Co^2+^	780.56			
		2p_1/2_	Co^3+^	794.34			
			Co^2+^	795.77			
	O 1s	O_lattice_		529.57			2.02
		O_ad_		530.48			
		H_2_O_ad_		533.08			
	C 1s			284.3			
LSM@Co_3_O_4_-20	Co	2p_3/2_	Co^3+^	779.53	0.68		
			Co^2+^	780.84			
		2p_1/2_	Co^3+^	794.58			
			Co^2+^	796.00			
	Mn	2p_3/2_	Mn^3+^	641.31		0.77	
			Mn^4+^	643.34			
		2p_1/2_	Mn^3+^	652.93			
			Mn^4+^	655.19			
	O 1s	O_lattice_		529.33			2.39
		O_ad_		530.40			
		H_2_O_ad_		532.44			
	C 1s			284.3			

## Data Availability

The data presented in this study are available in manuscript.
